# Clinicopathologic features, prognostic factors, and outcomes of visceral sarcomas: A retrospective 12-year single-center study

**DOI:** 10.3389/fonc.2022.1029913

**Published:** 2022-11-07

**Authors:** Songwei Yang, Zhichao Liao, Ting Li, Haotian Liu, Zhiwu Ren, Haixiao Wu, Jun Zhao, Sheng Teng, Ruwei Xing, Yun Yang, Jilong Yang

**Affiliations:** ^1^ Departments of Bone and Soft Tissue Tumor, Chongqing University Cancer Hospital, Chongqing, China; ^2^ Departments of Bone and Soft Tissue Tumor, Tianjin Medical University Cancer Institute & Hospital, Tianjin, China; ^3^ National Clinical Research Center of Cancer, Tianjin Medical University Cancer Institute & Hospital, Tianjin, China

**Keywords:** soft tissue sarcoma, visceral sarcoma, prognostic factor, outcome, Surveillance, Epidemiology, End Results database

## Abstract

**Background:**

Visceral sarcomas are a rare form of soft tissue sarcoma. This study aimed to evaluate the survival and prognostic factors and effective treatments for visceral sarcomas.

**Methods:**

All patients with visceral sarcoma referred to our center between January 2010 and December 2021 were retrospectively analyzed. The Kaplan-Meier method and a log-rank test were used for survival analysis.

**Results:**

A total of 53 patients with visceral sarcoma were analyzed in this study with the median age at diagnosis of 57 (range, 24-77) years. Among them, 37 (69.8%) and 16 (30.2%) patients had localized and metastatic diseases at the initial presentation, respectively, and 44 patients underwent surgical resection. The median follow-up, event-free survival (EFS) and overall survival (OS) were 63.0 (range, 2-130), 42.0 months (95% confidence interval [CI] 10.879-73.121) and 45.0 months (95% CI 9.938-80.062), respectively. The 5-year EFS and OS rates were 44% and 46%, respectively. Univariate analysis of prognostic indicators illustrated that metastasis at presentation, surgery, surgical margin and the types of surgery were significantly associated with OS and EFS. In this study, combined chemotherapy or radiotherapy had no effects on EFS and OS.

**Conclusion:**

Primary visceral sarcoma is an uncommon and aggressive malignant tumor with a higher rate of local recurrence. In the largest cohort of visceral sarcomas in China to date, we identified metastases at presentation, surgery, surgical margin, and the types of surgery as independent predictors of survival. The combination of chemotherapy and radiotherapy did not affect survival.

## Introduction

Soft tissue sarcomas (STS) are extremely uncommon tumors, accounting for only approximately 1% of all adult cancers (1). In the United States, an estimated 13,460 new cases were diagnosed and 5,350 deaths were caused by STS in 2021 (2). In China, approximately 39,900 new STS cases were reported in 2014, and the crude incidence rate was 2.91 per 100,000 (3). STS is a diverse collection of malignant tumors that includes >100 distinct histologic subtypes (4). The above-mentioned tumors are most frequently observed in the retroperitoneum, extremities, trunk, and head and neck area (1). Visceral sarcomas except for gastrointestinal stromal tumors (GIST) rarely occur, and only a few research reports have been available, which were mainly individual case reports (5, 6). Currently, no published research has investigated a significant number of patients with visceral sarcomas to the best of our knowledge, and therefore, little is known about the natural course, therapeutic approaches, and prognostic factors of these tumors. This study aimed to elucidate the clinical characteristics, therapeutic strategies, and prognostic factors of 53 patients with this disease, who received treatment between January 2010 and December 2021 at Tianjin Medical University Cancer Institute and Hospital.

## Materials and methods

### Patients

Patients with visceral sarcomas who were diagnosed and treated from January 2010 to December 2021 at Tianjin Medical University Cancer Institute and Hospital were enrolled. The diagnosis of visceral sarcoma is confirmed by a postoperative specimen or biopsy. Uterine sarcomas and GISTs were excluded. All patients underwent surgery, radiotherapy, chemotherapy, targeted therapy, radiofrequency ablation, or other palliative treatments in our hospital. Approval of the present study was granted by the Ethics Committee of Tianjin Cancer Institute. Clinicopathologic data were collected, including sex, age at diagnosis, tumor size, tumor site, metastases at appearance, surgical modality, resection margins, radiotherapy, chemotherapy, targeted therapy, radiofrequency ablation, and patient outcome. We decided to only include patients who had complete clinical information for therapy and follow-up. We also included two cases of postoperative death due to severe intraperitoneal infection and intestinal obstruction in order to reduce bias. In addition, we excluded four cases of pediatric patients due to the natural history and disease biology of pediatric sarcomas are different than adult ones. A total of 53 patients were available for analysis in our investigation.

We also analyzed patients with visceral sarcoma in the Surveillance, Epidemiology, and End Results (SEER) database (http://seer.cancer.gov/) from 2003 to 2015. Visceral sarcoma incidents were discovered utilizing the International Classification of Diseases for Oncology (ICD-O-3) topographic codes. Data were retrieved using the SEER*Stat software (https://seer.cancer.gov, version 8.3.9). A total of 6,147 eligible patients were identified from the SEER database.

### Follow-up

During the first 3 years, patients were followed up after every 3 months, every 6 months for 4-5 years, and then once a year thereafter according to the National Comprehensive Cancer Network (NCCN) (7) and the Chinese Society of Clinical Oncology (CSCO) guidelines for soft tissue sarcoma. Each follow-up included physical examinations, local B-ultrasonography, chest CT scans, and routine laboratory tests. Moreover, bone scans were conducted every 6 months for the first 5 years, and then once a year thereafter.

### Response criteria and outcomes evaluated

The Response Evaluation Criteria in Solid Tumors (RECIST) (version: 1.1) was utilized to evaluate the responsiveness of the tumor to therapy (8). A complete response (CR) was described as the total elimination of all lesions for a minimum period of 4 weeks after starting the therapy. A partial response (PR) was characterized by a decrease in the overall diameter of the target lesions by ≥30% when compared to the baseline measurement. When the total number of pretreatment lesion products increased by >20% or when a new illness appeared, progressive disease (PD) was considered to have occurred. The lack of either PD or PR was regarded as a stable disease (SD). Beginning from the initial diagnosis to the time of death attributable to any factor or the completion of the follow-up period, overall survival (OS) was calculated for each patient (censored data). Event-free survival (EFS) was defined as the time from initial management to tumor progression, relapse, or death.

### Statistical analysis

Statistical analyses were carried out using SPSS (version: 22.0) (IBM, Armonk, NY, USA). EFS and OS were estimated using the Kaplan-Meier method. Potential risk variables, including sex, age, tumor site, tumor size, metastases, surgical, and resection margins, were examined by univariate analysis. To compare curves from a univariate study, the log-rank test was utilized. EFS and OS were both evaluated using the Cox proportional hazards model, which was applied to determine independent prognostic variables. *p*-value < 0.05 was considered to indicate statistical significance.

## Results

### Patients’ clinical and pathologic features

**Figure 1:**
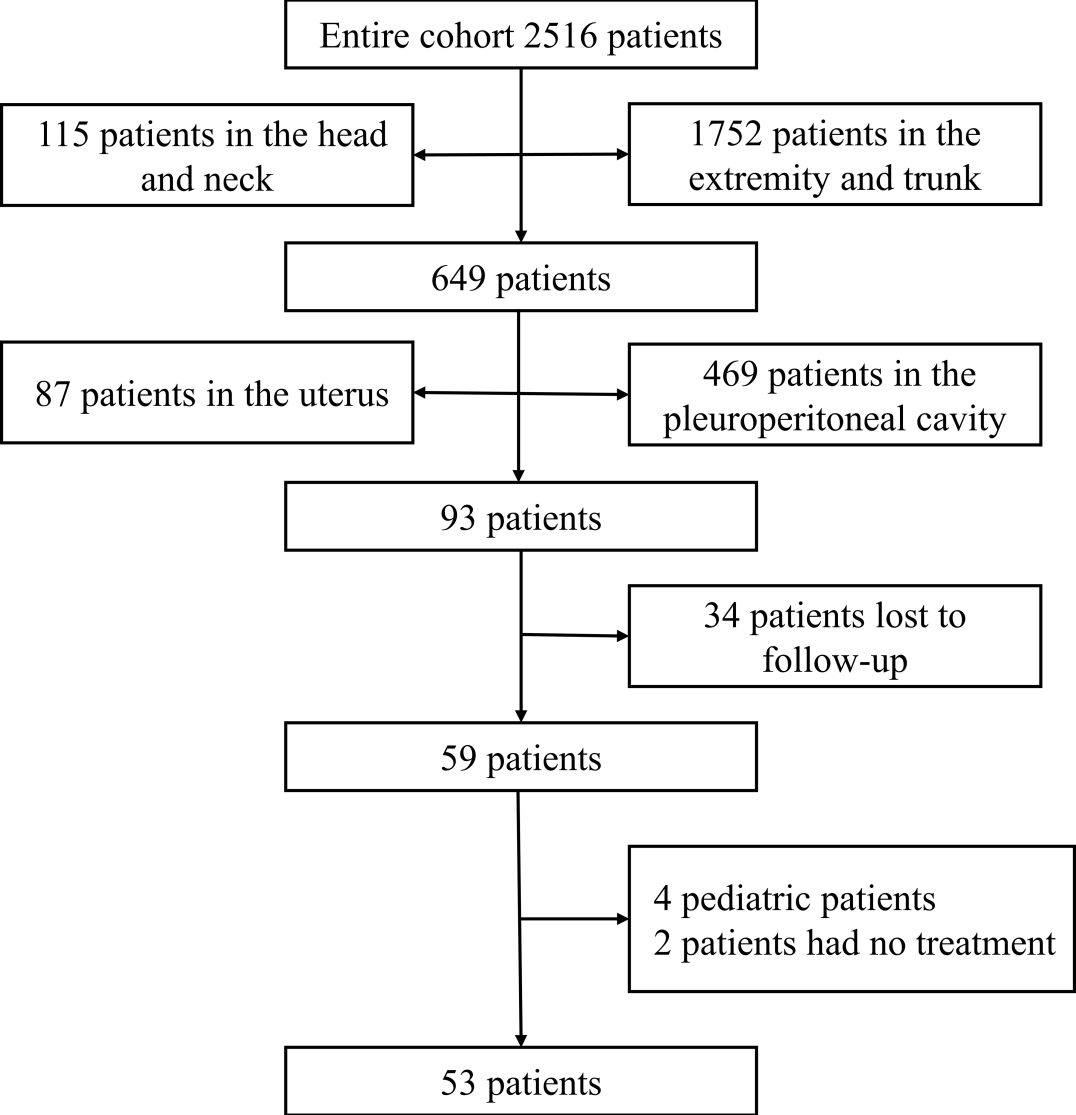
Flowchart of the selection of study patients.

A total of 2516 cases of soft tissue sarcomas at all anatomical sites (except GISTs) were enrolled from January 2010 to December 2021. Sarcomas arising at the extremities or trunk, retroperitoneum, head and neck, and uterine corpus were excluded (n = 2423). Over 12 years, 93 patients with visceral sarcomas were hospitalized at the Tianjin Medical University Cancer Institute and Hospital. Thirty-four patients were lost to follow-up or had missing data. Four patients were pediatric, and two patients did not receive any therapy. These 40 patients were excluded from the final analysis (**Figure 1**). We enlisted a total of 53 patients with visceral sarcomas comprising 28 females and 25 males aged between 24 and 77 years with the median age being 57 years. 11 patients (20.75%) presented with primary sites in the liver, whereas 11 individuals (20.75%) presented with primary sites in the lung. At the time of presentation, 37 patients (69.81%) exhibited local disease, while 16 patients (30.19%) exhibited metastatic lesions. The median tumor size of 44 patients was 13.35 cm (range, 0.7-26), and the others were of unknown size. The most commonly represented pathological types were leiomyosarcoma 14 (26.42%), undifferentiated pleomorphic sarcoma (UPS) 9 (16.98%), liposarcoma 6 (11.32%), and undifferentiated sarcoma (US) 6 (11.32%). **Table 1** lists the clinical features of patients.

**Table 1:** Clinicopathological features of visceral sarcomas.

Clinical parameters	Total (53)	%
Age (year)	24-77(57)	
Range (median)		
<57	25	47.17%
≥57	28	52.83%
Sex		
Female	28	52.83%
Male	25	47.17%
Tumour location		
Liver	11	20.75%
Kidney	4	7.55%
Lung	11	20.75%
cStomach	5	9.44%
Bowel	11	20.75%
Oesophagus	3	5.66%
Pancreas	5	9.44%
Bladder	2	3.77%
Atrium	1	1.89%
Metastases		
Yes	16	30.19%
No	37	69.81%
Surgery		
Yes	44	83.02%
No	9	16.98%
Chemotherapy		
Yes	17	32.08%
No	36	62.92%
Radiotherapy		
Yes	9	16.98%
No	44	83.02%
Tumor size		
≤5cm	18	33.96%
>5cm	26	49.06%
Unknown	9	16.98%
Surgical margin		
Negative	37	69.81%
Positive	16	30.19%
Metastatic sites		
Lymph node	3	5.66%
Distant	12	22.64%
Both	1	1.89%
Pathological type		
UPS	9	16.98%
Liposarcoma	6	11.32%
Leiomyosarcoma	14	26.42%
Synovial sarcoma	1	1.89%
IMT	3	5.66%
UES	2	3.77%
MSFB	2	3.77%
MPNST	4	7.54%
ASTS	1	1.89%
US	6	11.32%
PNET	1	1.89%
Fibrosarcoma	1	1.89%
Others	3	5.66%

UPS, undifferentiated pleomorphic sarcoma; IMT, inflammatory myofibroblastic tumor; UES, undifferentiated embryonal sarcoma; MPNST, malignant peripheral nerve sheath tumor; ASTS, alveolar soft tissue sarcoma; US, undifferentiated sarcoma; MSFT, malignant solitary fibrous tumor; PNET, primitive neuroectodermal tumor.

### Therapy, recurrence, and metastases

Local control treatments were performed with surgery alone, surgery plus radiotherapy, or radiotherapy alone. Resections were performed in all primary diseases but nine patients. In 24 patients (45.28%), a large resection involving negative surgical margins was accomplished. Then, 13 (24.53%) patients underwent marginal resection with negative surgical margins. There were seven patients of intralesional resection with a positive margin, nine patients only received biopsy, 17 patients (32.08%) had undergone combined chemotherapy, 16 of them underwent adjuvant chemotherapy, and three neoadjuvant chemotherapy combined with adjuvant chemotherapy. One patient with primitive neuroectodermal tumor (PNET) was treated with vincristine 1.2 mg/m^2^/day (Day 1), doxorubicin 30 mg/m^2^/day (Day 1-2), cyclophosphamide 1200 mg/m^2^/day (Day 1) (VAC) alternating with ifosfamide 1800 mg/m^2^/day (Day 1-5), etoposide 100 mg/m^2^/day (Day 1) (IE), and mesna equivalent dose divided into three doses, including one before and two after ifosfamide administration for 5 days. Twelve cycles of chemotherapy were scheduled, with each treatment delivered every three weeks. Three patients received doxorubicin 30 mg/m2/day (Day 1-2), dacarbazine 400 mg mg/m2/day (Day 1-5), ifosfamide 1800 mg/m2/day (Day 1-5) with mesna (MIAD). The chemotherapeutic regimens of other patients with visceral nonrhabdomyosarcoma soft tissue sarcomas were doxorubicin 30 mg/m^2^/day (Day 1-2), ifosfamide 1800 mg/m^2^/day (Day 1-5) with mesna (AI). Five patients received second-line treatment after failure of first-line chemotherapy, including gemcitabine plus docetaxel or ifosfamide and etoposide. 7 patients received post-surgical radiotherapy at the primary site, including 4 postoperative chemoradiotherapy and 3 postoperative radiotherapies alone. One patient who could not be surgically resected at the time of diagnosis received chemoradiotherapy, and another only received radiotherapy. Five patients had received targeted therapy, comprising three who have progressed after other treatment and two who could not be resected received targeted therapy immediately after diagnosis. In addition, two patients who had failed multiline therapy received immunotherapy. **Table 2** summarizes the particulars of the treatment plan.

**Table 2:** Treatment and recurrence details of patients.

	Total (53)	%
Types of surgery		
Wide resection	24	45.28%
Marginal resection	13	24.53%
Intralesional resection or biopsy	16	30.19%
Primary therapy		
Surgery	27	50.94%
Surgery + CT	6	11.32%
Surgery + CT + RT	2	3.77%
Surgery + CT + TT	1	1.89%
Surgery + CT + RT + RA	1	1.89%
Surgery + CT + IT	1	1.89%
Surgery + CT + RT + TT + IT	1	1.89%
Surgery + RT	2	3.77%
Surgery + RT + IT	1	1.89%
Surgery + RA	1	1.89%
Surgery + RA + TT	1	1.89%
CT	4	7.55%
CT + RT+RA	1	1.89%
RT	1	1.89%
TT	2	3.77%
RA	1	1.89%
Tumour recurrence		
Local	15	28.30%
Distant	4	7.55%
Both	4	7.55%

CT, chemotherapy; RT, radiotherapy; TT, targeted therapy; IT, immunotherapy; RA, radiofrequency ablation.

Local recurrence and metastasis occurred in 23 (52.27%) patients postoperatively, including 6 patients with lymph nodes and local metastases at presentation (**Table 2**). The local recurrence rate was 43.40%. Of patients with local recurrence and metastasis (23), 18 (78.26%)had not previously received adjuvant radiotherapy or chemotherapy.

### Survival analysis

**Figure 2:**
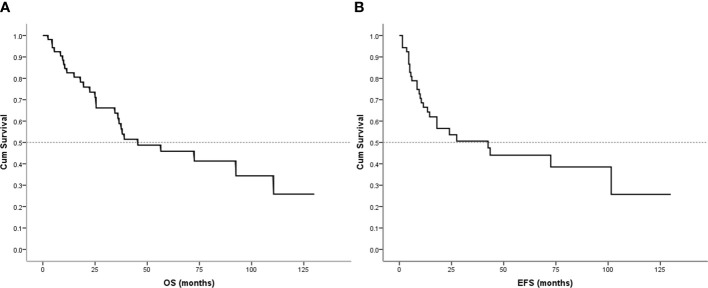
Survival curves for OS and EFS. **(A)** Survival curves for OS. **(B)** Survival curves for EFS.

**Figure 3:**
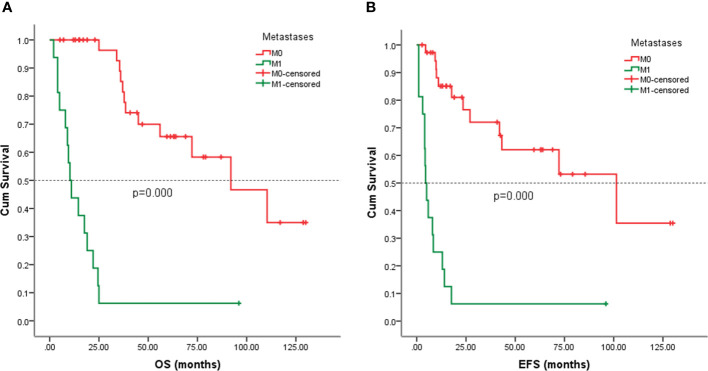
Kaplan-Meier analysis of OS and EFS for patients with or without metastasis at presentation. **(A)** Kaplan-Meier analysis of OS for patients with or without metastasis at presentation (p=0.000). **(B)** Kaplan-Meier analysis of EFS for patients with or without metastasis at presentation (p=0.000).

**Figure 4:**
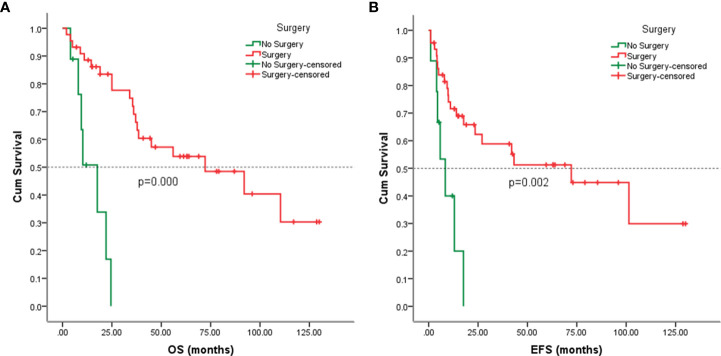
Kaplan-Meier analysis of OS and EFS for patients with or without surgery. **(A)** Kaplan-Meier analysis of OS for patients with or without surgery (p=0.000). **(B)** Kaplan-Meier analysis of EFS for patients with or without surgery (p=0.002).

**Figure 5:**
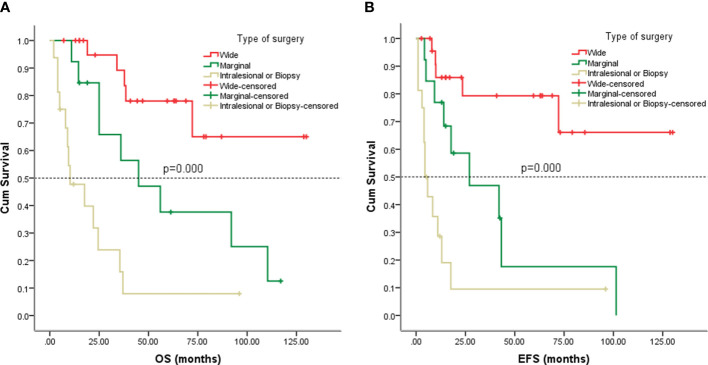
Kaplan-Meier analysis of OS and EFS for patients with different types of surgery. **(A)** Kaplan-Meier analysis of OS for patients with different types of surgery (p=0.000). **(B)** Kaplan-Meier analysis of EFS for patients with different types of surgery (p=0.000).

**Figure 6:**
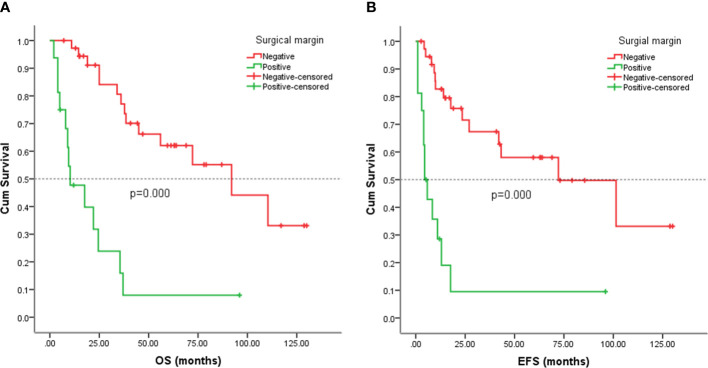
Kaplan-Meier analysis of OS and EFS for patients with positive or negative surgical margin. **(A)** Kaplan-Meier analysis of OS for patients with positive or negative surgical margin (p=0.000). **(B)** Kaplan-Meier analysis of EFS for patients with positive or negative surgical margin (p=0.000).

Follow-up with patients was carried out until they died or until December 2021, whichever came first. The follow-up duration for all patients ranged from 2 to 130 months with the median duration being 63.0 months. At the time of the final follow-up, a sum of 26 patients (49.06%) had passed away. All deaths were due to disease progression except two cases of postoperative death due to severe intraperitoneal infection and intestinal obstruction and two patients died of other illnesses. 19 individuals (35.85%) were still alive and free of illness; eight (15.09%) patients survived with tumors. The anticipated three- and five-year OS rates for all patients were 59% and 46%, respectively (**Figure 2A**). The median OS was 45.0 months (95% CI 9.938-80.062). The anticipated three- and five-year EFS for all patients were 51% and 44%, respectively (**Figure 2B**). The median EFS was 42.0 months (95% CI 10.879-73.121). The five-year OS rate for M0 patients was 66%, significantly higher than that for patients with M1 (6%) (*p* = 0.000) (**Figure 3A**). The five-year EFS rate for M0 patients was 62%, in contrast with 6% for patients diagnosed with M1, *p* = 0.000 (**Figure 3B**). As opposed to patients without surgery, we identified a favorable five-year OS rate compared to those who underwent surgical operations (five-year OS rate: 54% vs 0%, *p* = 0.000) (**Figure 4A**). Five-year EFS was considerably improved in patients who underwent surgery compared to those who did not (five-year EFS rate: 51% vs 0%, *p* = 0.002) (**Figure 4B**). Patients with wide tumor resection had the most favorable survival: 65% surviving at 10 years and 78% at 5 years (**Figure 5A**). For patients who underwent a marginal resection, the five-year OS rate was 38% (*p* = 0.022). While compared to patients who had undergone wide resection, the 5-year OS rate for patients with intralesional resection or biopsy was 8% (*p* = 0.000). For patients whose intervention involved a wide or marginal resection, 5-year EFS rates were 79% and 18% (*p* = 0.005), respectively (**Figure 5B**). The five-year EFS rate for patients with intralesional resection or biopsy was only 10%(*p* = 0.000). In addition, we also examined the relationship between surgical margin and prognosis. When compared to patients with positive surgical margins, those with negative surgical margins exhibited greater survival (five-year EFS rate: 58% vs 10%, *p* = 0.000, 5-year OS rate: 62% vs 8%, *p* = 0.000) (**Figures 6A, B**).

**Figure 7:**
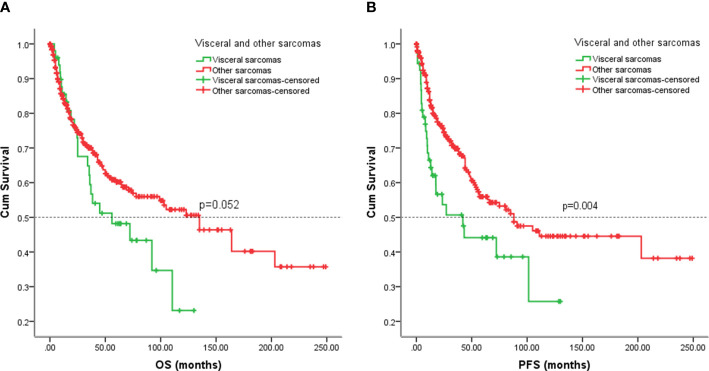
Kaplan-Meier analysis of OS and EFS for patients with visceral and other sarcomas. **(A)** Kaplan-Meier analysis of OS for patients with visceral and other sarcomas (p=0.052). **(B)** Kaplan-Meier analysis of EFS for patients with visceral and other sarcomas (p=0.004).

To compare with other sarcomas, we analyzed partial data on non-visceral sarcomas from Tianjin Medical University Cancer Hospital, including 9 leiomyosarcomas, 277 osteosarcoma, 146 MPNST, and 29 synovial sarcomas. Patients with other sarcomas did not have a favorable five-year OS rate compared to patients with visceral sarcomas, (five-year OS rate: 60% vs. 46%, *p =* 0.052) (**Figure 7A**). The five-year EFS rate for patients with other sarcomas was 56% in contrast with 44% for patients with visceral sarcomas (*p* = 0.004) (**Figure 7B**).

**Figure 8:**
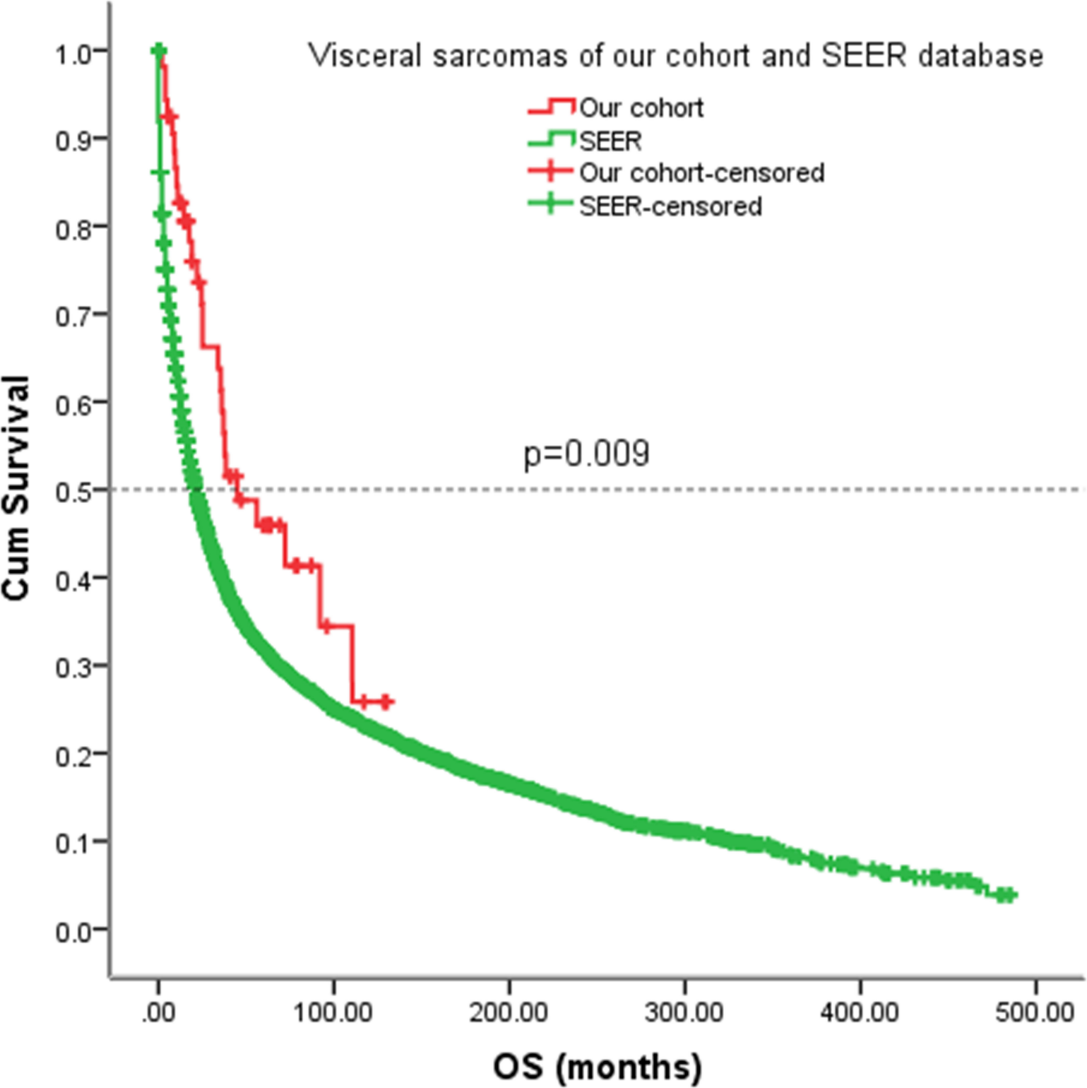
Kaplan-Meier analysis of OS for patients with visceral sarcomas of our cohort and SEER database.

As the information on EFS cannot be obtained from the SEER database, we analyzed the OS of our patients with the SEER cohort. For our patients with visceral sarcomas, the five-year OS rate was 46%. However, the five-year OS rate in the SEER cohort was 32% (*p* = 0.009) (**Figure 8**).

**Figure 9:**
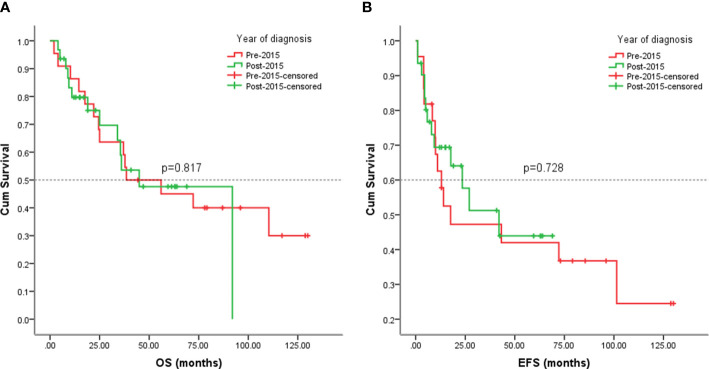
Kaplan-Meier analysis of OS and EFS for patients diagnosed with visceral sarcomas pre-2015 and post-2015. **(A)** Kaplan-Meier analysis of OS for patients diagnosed with visceral sarcomas pre-2015 and post-2015 (p=0.817). **(B)** Kaplan-Meier analysis of EFS for patients diagnosed with visceral sarcomas pre-2015 and post-2015 (p=0.728).

Since this study lasted for 10 years, we would like to know whether patients were responding better in recent years (after 2015) due to advances in sarcoma treatment. No significant differences in EFS (*p* = 0.728) and OS (*p* = 0.817) were observed among patients in the early and late eras (**Figure 9**).

**Figure 10:**
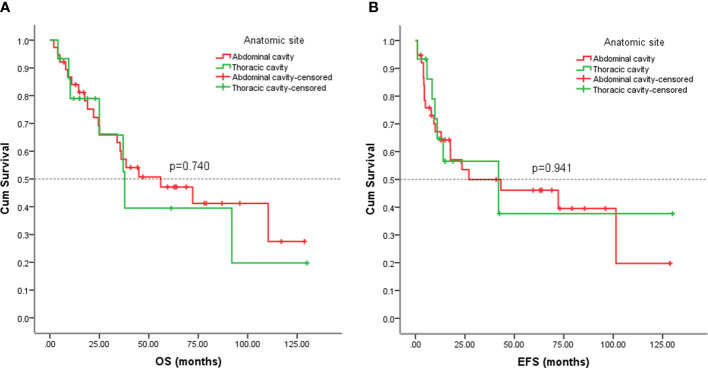
Kaplan-Meier analysis of OS and EFS for patients with visceral sarcoma in the abdominal and thoracic cavities. **(A)** Kaplan-Meier analysis of OS for patients with visceral sarcoma in the abdominal and thoracic cavities (p=0.740). **(B)** Kaplan-Meier analysis of EFS for patients with visceral sarcoma in the abdominal and thoracic cavities (p=0.941).

The 53 cases of our study, 38(71.70%) arose in the abdominal cavity, and the remainder 15(28.30%) in thoracic cavity. We also analyzed the outcomes of visceral sarcomas in different anatomic sites. However, the EFS (*p* = 0.941) and OS (*p* = 0.740) did not differ profoundly among the different anatomic sites (**Figure 10**).

### Multivariable and univariable analysis for survival

Multivariate and univariate analyses of the effects of associated prognostic variables on OS are shown in **Table 3**. In univariate analysis, no metastasis at presentation (p = 0.000), surgery (p = 0.000), wide resection (p = 0.000), and negative surgical margin (p = 0.000) were correlated with a favorable OS. A multivariable analysis showed that metastasis at presentation (HR= 10.881; 95% CI= 4.662–25.162; *p* =0.017) and surgical style (HR=2.026; 95% CI=1.024-4.011; *p* = 0.043) were correlated with OS. In univariate analysis, metastasis at presentation (*p* = 0.000), surgery (*p* = 0.004), types of surgery (*p* = 0.000), and surgical margin (*p* = 0.000) were correlated with EFS. In multivariate analysis, metastasis at presentation (HR=8.509; 95% CI=3.760-19.252; *p* =0.000) was unfavorable prognostic factor to EFS. **Table 4** depicts univariate and multivariate analyses of the impact of associated prognostic variables on EFS.

**Table 3:** Multivariable and univariate analyses of the OS in patients with visceral sarcomas.

Clinical parameters	Univariate analysis		Multivariate analysis	
	HR (95% CI)	*P*-value	HR (95% CI)	*P*-value
Age (year)				
<57	Reference			
≥57	1.176 (0.549-2.519)	0.677		
Sex				
Female	Reference			
Male	1.484 (0.689-3.193)	0.313		
Metastases				
No	Reference		Reference	0.000
Yes	10.831 (4.662-25.162)	0.000	10.881(4.662–25.162)	
Tumour size				
<5cm	Reference			
≥5cm	1.718 (0.982-3.005)	0.058		
Unknown	–	–		
Surgery				
No	Reference			
Yes	0.119 (0.040-0.351)	0.000		
Chemotherapy				
No	Reference			
Yes	1.634 (0.740-2.698)	0.225		
Radiotherapy				
No	Reference			
Yes	0.749(0.278-3.610)	0.568		
Surgical margin				
Negative	Reference			
Positive	6.346(2.835-14.203)	0.000		
Type of surgery				
Wide resection				
Marginal resection	Reference			
Intralesional resection or biopsy	3.410 (2.028-5.733)	0.000		
Year of diagnosis				
Pre-2015	Reference			
2015+	1.097(0.498-2.420)	0.818		
Anatomic site				
Abdominal cavity	Reference			
Thoracic cavity	1.158(0.487-2.755)	0.740		

HR, hazard ratio; CI, confidence interval.

**Table 4:** Univariable and multivariable analyses of the EFS in patients with visceral sarcomas.

Clinical parameters	Univariate analysis		Multivariate analysis	
	HR (95% CI)	*P*-value	HR (95% CI)	*P*-value
Age (year)				
≤57	Reference			
>57	1.315 (0.614–2.817)	0.481		
Sex				
Female	Reference			
Male	1.423 (0.663–3.054)	0.365		
Metastases				
No	Reference	0.000	Reference	0.000
Yes	8.509 (3.760–19.252)		8.509(3.760–19.252)	
Tumour size				
≤5 cm	Reference			
>5 cm Unknown	1.703 (0.988–2.935) -	0.055 -		
Surgery				
No	Reference			
Yes	0.246 (0.096–0.631)	0.004		
Chemotherapy				
No	Reference			
Yes	1.541 (0.700–3.389)	0.282		
Radiotherapy				
No	Reference			
Yes	1.257 (0.471–3.356)	0.648		
Surgical margin				
Negative	Reference			
Positive	5.853 (2.605–13.151)	0.000		
Type of surgery				
Wide resection				
Marginal resection	Reference			
Intralesional resection or biopsy	3.006 (1.982–5.514)	0.000		
Year of diagnosis				
Pre-2015	Reference			
2015+	0.870 (0.395–1.915)	0.729		
Anatomic site				
Abdominal cavity	Reference			
Thoracic cavity	0.968(0.405-2.311)	0.941		

## Discussion

Visceral sarcoma is an exceptionally rare tumor. At present, our knowledge of these malignant tumors is limited to isolated case reports and small series reports. Therefore, no specific management guidelines have been established. We report 53 cases of primary visceral sarcoma diagnosed in our hospital in the past 12 years. The present study is the first to describe the clinicopathologic characteristics and identify prognostic factors in this rare class of malignancies in China.

The predominant histologic subtype in the study group was leiomyosarcoma (26.42%) and UPS (16.98%). This distribution is different from the extremities and trunk. Most limb and trunk sarcomas are UPS, whereas most visceral sarcomas are leiomyosarcomas and liposarcoma (9–12). This distribution is consistent with all adult soft tissue sarcomas (1). Age at diagnosis has been repeatedly reported in different series, with a median value of 52-62 years (5, 13, 14). The median age of our patients was 57 years during diagnosis, which is consistent with earlier studies. About 52.83% of patients were women; however, no significant sex predilection was observed in our cohort and in other reports (9).

In soft tissue sarcoma, the tumor size is thought to be a significant prognostic marker (15–17). In our cohort, patients with tumors of <5 cm tended to have a longer OS, and no statistical significance was found (*p* = 0.058). Since our series included patients with metastatic and unresectable diseases, only 44 patients with complete size data were analyzed, and the sample size was small; thus, we could not draw a conclusion. “Soft Tissue Sarcoma of the Abdomen and Thoracic Visceral Organs” is a new chapter in the eighth edition of the AJCC Cancer Staging Manual (18). No prognostic staging grouping is offered, although the recommended primary tumor (T) staging is based on organ multifocality and confinement rather than the maximum tumor size. This may indicate that the size of visceral sarcoma is not an important prognostic factor, which is different from the trunk and extremities. The conventional sarcoma staging system is not suitable for visceral sarcomas, and some studies support this view (14). Nevertheless, according to broad series from the SEER database, established prognostic variables, including the incidence of metastatic illnesses, histologic grade, and tumor size, are also definitely applicable for visceral sarcoma (9). Further evidence is needed to demonstrate whether tumor size is relevant to visceral sarcomas.

Lymph node metastasis (LNM) in soft tissue sarcoma is a relatively rare event but is associated with worse survival (14, 19, 20). Recently, a large series of analyses reported that the incidence of LNM in sarcomas of the intrathoracic (5.3%) and intraabdominal/retroperitoneal (5.1%) sites more frequently than those of the trunk or extremities (2%) (21, 22). In our series, four patients (7.55%) had LNM, representing the highest rate.

Patients with metastases at the time of diagnosis exhibited a poorer prognosis in the previously described series (15, 23, 24). Similarly, patients with early metastases had a considerably worse outcome in the present study, both in terms of EFS and OS. Compared to the sarcomas of the trunk and extremities, visceral sarcomas have a high likelihood of presenting metastasis (9).

In previous studies, complete resection was considered to be among the most crucial prognostic markers (5, 14, 24). In the present study, 44 patients underwent surgical resection, including 35 patients with localized diseases and 9 patients with metastatic diseases. In our cohort, surgery was significantly associated with OS and EFS (**Figure 4**), although the inclusion of patients with metastatic disease in this analysis may compromise the results. Previous studies have illustrated the significance of wide surgical margins for good survival (16, 24). Fariba et al. (23) also demonstrated children and adolescents with visceral nonrhabdomyosarcoma soft tissue sarcomas treated with a wide or marginal excision exhibited favorable control of the local tumor and improved OS and EFS. In our cohort, patients who underwent wide excision had better survival than those who underwent marginal resection and intralesional resection or biopsy. The local recurrence rate of visceral tumors and retroperitoneal soft tissue sarcomas was higher (10, 23, 25). A total of 23 (52.27%) patients have experienced local recurrence or metastasis in this study. Tumors sited in the viscera were found to be bigger, with a higher likelihood of invasiveness, and a closer distance to vital structures and organs, which attribute to the difficulty of complete surgical resection. This might be one of the contributing factors to the increased risk of local recurrence of visceral sarcomas.

The effectiveness of chemotherapy in this case series is difficult to assess because of the inclusion of a wide range of diverse histologic subgroups and a large number of dissimilar chemotherapy regimens. Three of four children with UPSL received neoadjuvant chemotherapy and a decrease in the tumor volume following chemotherapy and no detectable malignant cells were found in resected specimens, and a long-time complete remission was achieved after adjuvant chemoradiotherapy. The other case was referred to our hospital postoperatively and only received postoperative adjuvant radiotherapy and chemotherapy. Unfortunately, the patient developed recurrence and metastasis and eventually died. Although it is impossible to draw a definite conclusion from such a small sample size, these data still show that neoadjuvant chemotherapy performs an integral function in the prognosis of children with UPSL. Moreover, our results are supported by previous studies with small samples (26, 27), and this question deserves further investigation if a larger patient dataset is available. The four cases of pediatric patients were eliminated due to the natural history and disease biology of pediatric sarcomas are different than adult ones. Similarly, indications and doses of radiotherapy were not normalized in this series, making it difficult to determine whether the application of radiotherapy affected the rate of local control formation. Notwithstanding the above drawbacks and the fact that the overall number of patients who received each therapy was limited, the local rate of failure of patients who underwent resection and adjuvant radiotherapy was not distinguishable from that of patients who underwent surgical procedure alone, thereby highlighting the significance of surgical resection in treating these patients.

Visceral and other sarcomas seem to have different outcomes. In our study, visceral tumors had a high likelihood of exhibiting a poor EFS, whereas sarcomas in other sites had similar OS to visceral sarcomas. The majority of patients with visceral tumors cannot be indicated for extensive local or marginal resection, which is an integral part of local treatment (23). Visceral sarcoma is large in size and adjacent to important structures and organs, resulting in difficult complete surgical resection and a high local recurrence rate, which has been improved in retroperitoneal soft tissue sarcomas (10, 25, 28, 29).

To compare survival data in our cohort with others, we analyzed patients with visceral sarcoma in the SEER database. The OS in our cohort seems to be better than the SEER cohort; however, the small cases in our cohort make it difficult to draw an effective conclusion.

The results of our investigation revealed the few advancements in the therapy for visceral sarcomas, with patients diagnosed post-2015 faring no differently in contrast with those who were diagnosed before 2015. While the general treatment of soft tissue sarcoma underwent little improvement in the past few years, innovations in imaging, surgical technology, and radiotherapy seem to have a minor influence on the prognosis of these patients, which poses a special challenge to our traditional treatment.

In summary, the present study represents the first population-level study of patients with visceral sarcoma in China. We identified metastases at presentation and types of surgery as independently associated with survival. Furthermore, our study revealed that the use of adjuvant chemotherapy in conjunction with adjuvant radiation does not increase the OS or EFS rates. It is well known that retrospective observational studies have more confounding factors due to the lack of randomization, and due to the extremely low incidence of visceral sarcoma, the small sample size of this study makes it impossible to draw reliable conclusions from subgroup analyses. Further studies with larger, prospective cohorts of these neoplasms are needed to answer important questions such as the prognostic value of tumor size or the role of adjuvant chemotherapy and to ensure the optimal, evidence-based management of patients with these rare tumors.

## Data availability statement

The raw data supporting the conclusions of this article will be made available by the authors, without undue reservation.

## Ethics statement

The studies involving human participants were reviewed and approved by Tianjin Medical University Cancer Institute & Hospital ethics committee. Written informed consent for participation was not required for this study in accordance with the national legislation and the institutional requirements.

## Author contributions

SY collected and analyzed data, written original draft and edited. ZL, TL, HL, ZR, HW, JZ, RX, ST, and YY collected and analyzed data. JY: Designed and administrated the project, and final responsibility for the decision to submit the manuscript for publication. All authors were actively involved in the preparation of this manuscript. All authors have read and approved the final manuscript.

## Funding

This work was partly supported by the Nature Science Foundation of Tianjin (grant number 16JCYBJC24100 to JY and18YFZCSY00550 to JY) and The Science & Technology Development Fund of Tianjin Education Commission for Higher Education (grant number 2021KJ199 to TL).

## Acknowledgments

The authors thank Bullet Edits Limited for language editing of the manuscript.

## Conflict of interest

The authors declare that the research was conducted in the absence of any commercial or financial relationships that could be construed as a potential conflict of interest.

## Publisher’s note

All claims expressed in this article are solely those of the authors and do not necessarily represent those of their affiliated organizations, or those of the publisher, the editors and the reviewers. Any product that may be evaluated in this article, or claim that may be made by its manufacturer, is not guaranteed or endorsed by the publisher.

## Abbreviations

EFS, event-free survival; OS, overall survival; STS, soft tissue sarcomas; GIST, gastrointestinal stromal tumors; SEER, Surveillance, Epidemiology, and End Results; NCCN, National Comprehensive Cancer Network; CSCO, Chinese Society of Clinical Oncology; CR, complete response; PR, partial response; SD, stable disease; PD, progressive disease; UPS, undifferentiated pleomorphic sarcoma; IMT, inflammatory myofibroblastic tumor; UES, undifferentiated embryonal sarcoma; MPNST, malignant peripheral nerve sheath tumor; ASTS, alveolar soft tissue sarcoma; US, undifferentiated sarcoma; MSFT, malignant solitary fibrous tumor; PNET, primitive neuroectodermal tumor.
